# Smurf2 regulates stability and the autophagic–lysosomal turnover of lamin A and its disease‐associated form progerin

**DOI:** 10.1111/acel.12732

**Published:** 2018-02-05

**Authors:** Aurora Paola Borroni, Andrea Emanuelli, Pooja Anil Shah, Nataša Ilić, Liat Apel‐Sarid, Biagio Paolini, Dhanoop Manikoth Ayyathan, Praveen Koganti, Gal Levy‐Cohen, Michael Blank

**Affiliations:** ^1^ Laboratory of Molecular and Cellular Cancer Biology Azrieli Faculty of Medicine Bar‐Ilan University Safed Israel; ^2^ Department of Pathology The Galilee Medical Center Nahariya Israel; ^3^ Department of Pathology and Laboratory Medicine IRCCS Fondazione Istituto Nazionale dei Tumori Milan Italy

**Keywords:** autophagy, Hutchinson‐Gilford progeria syndrome, lamin A, progerin, Smurf2, ubiquitination

## Abstract

A‐lamins, encoded by the *LMNA* gene, are major structural components of the nuclear lamina coordinating essential cellular processes. Mutations in the *LMNA* gene and/or alterations in its expression levels have been linked to a distinct subset of human disorders, collectively known as laminopathies, and to cancer. Mechanisms regulating A‐lamins are mostly obscure. Here, we identified E3 ubiquitin ligase Smurf2 as a physiological regulator of lamin A and its disease‐associated mutant form progerin (LAΔ50), whose expression underlies the development of Hutchinson‐Gilford progeria syndrome (HGPS), a devastating premature aging syndrome. We show that Smurf2 directly binds, ubiquitinates, and negatively regulates the expression of lamin A and progerin in Smurf2 dose‐ and E3 ligase‐dependent manners. Overexpression of catalytically active Smurf2 promotes the autophagic–lysosomal breakdown of lamin A and progerin, whereas Smurf2 depletion increases lamin A levels. Remarkably, acute overexpression of Smurf2 in progeria fibroblasts was able to significantly reduce the nuclear deformability. Furthermore, we demonstrate that the reciprocal relationship between Smurf2 and A‐lamins is preserved in different types of mouse and human normal and cancer tissues. These findings establish Smurf2 as an essential regulator of lamin A and progerin and lay a foundation for evaluating the efficiency of progerin clearance by Smurf2 in HGPS, and targeting of the Smurf2–lamin A axis in age‐related diseases such as cancer.

## 1 INTRODUCTION

The nuclear lamina (NL) is an essential component of metazoan cells. In addition to conferring to the nucleus its shape and mechanical stability, NL is implicated in key nuclear functions including chromatin organization, transcription, DNA replication, and repair (Burke & Stewart, 2013; Dechat et al., 2008; Dittmer & Misteli, 2011).

A‐type lamins (lamin A, C, C2, and lamin AΔ10) are major structural components of the nuclear lamina. These lamins are encoded by a single gene–*LMNA* in humans (Lin & Worman, 1993; Machiels et al., 1996; Nakajima & Abe, 1995). Lamin A and lamin C (lamin A/C) are the major isoforms of A‐lamins that are expressed in somatic cells, whereas lamin C2 is uniquely expressed in the testis (Furukawa, Inagaki & Hotta, 1994). The minor lamin AΔ10 is also found in somatic cells, although its overall expression profile remains unclear (Burke & Stewart, 2013). Lamin C is synthesized directly from the *LMNA* gene, whereas lamin A is first produced as a precursor*—*prelamin A. Subsequently, prelamin A undergoes a few posttranslational modifications to generate the mature protein (Dechat, Adam, Taimen, Shimi & Goldman, 2010).

Mutations in the *LMNA* gene and/or alterations in its expression levels have been linked to a variety of distinct degenerative diseases, collectively known as the laminopathies, and to cancer (Burke & Stewart, 2013; Sakthivel & Sehgal, 2016).

**Figure 1:**
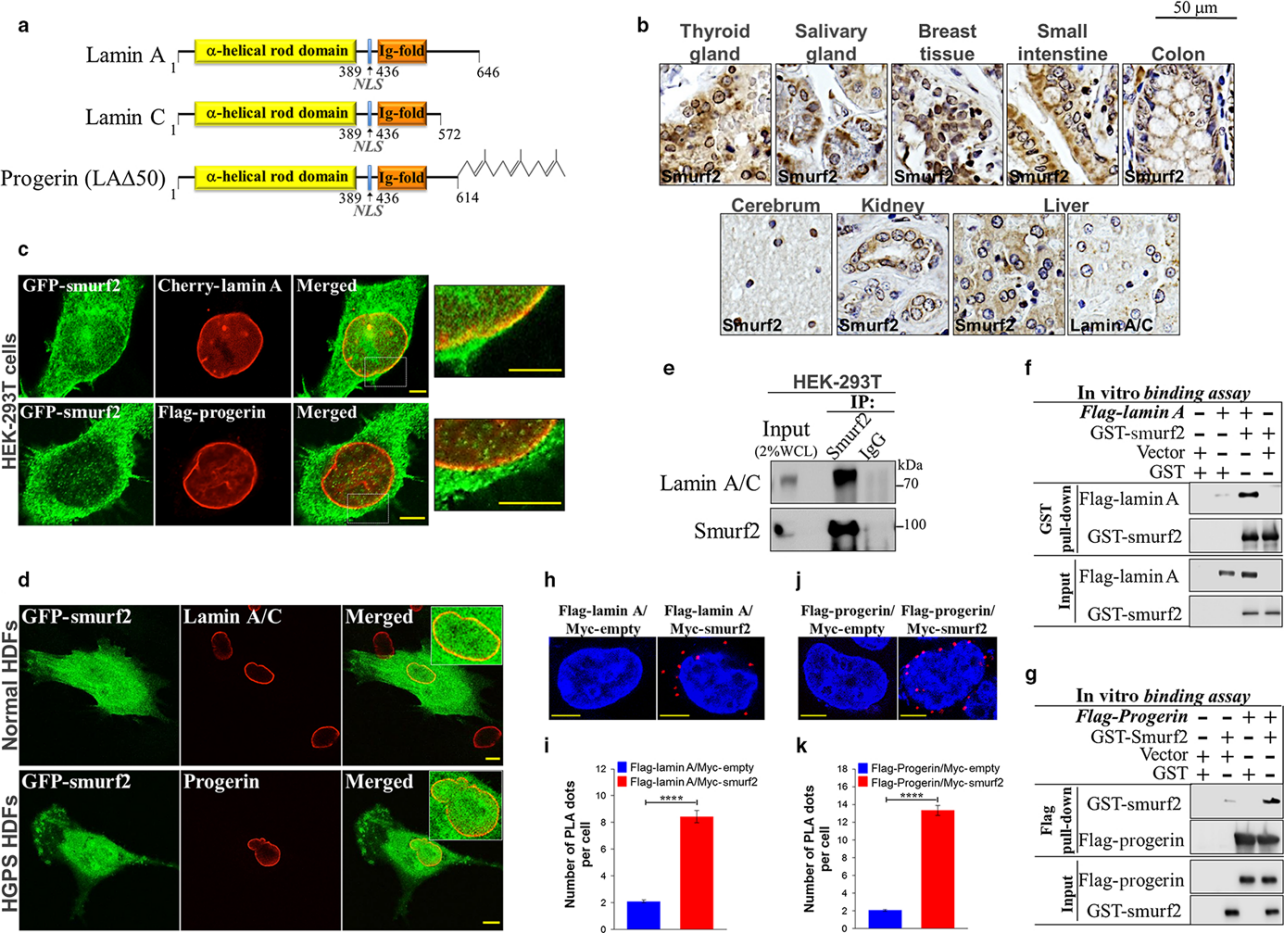
Smurf2 physically interacts with lamin A and its dominant mutant form progerin. (a) Schematic diagram of the structure of lamin A, lamin C, and progerin (LAΔ50). Progerin retains its C‐terminal CAAX motif that is stably farnesylated. NLS—nuclear localization signal. (b) Immunohistochemistry (IHC) analysis of Smurf2 expression and biodistribution in a panel of human normal tissues (FDA999m TMA). IHC staining of both Smurf2 and A‐lamins in liver tissues is also shown. (c) Confocal images showing co‐localization of GFP–Smurf2 with mCherry‐lamin A and Flag–progerin expressed in human HEK‐293T cells. Bars, 5 μm. (d) Co‐localization of GFP–Smurf2 with lamin A/C and progerin in normal and HGPS HDF cells. Bars, 10 μm. (e) Co‐IP analysis of endogenous Smurf2 and lamin A/C interaction in HEK‐293T cells. WCL, whole cell lysate. (f) In vitro binding assay showing a direct interaction between purified GST–Smurf2 and Flag–lamin A. (g) In vitro binding assay showing a direct interaction between Flag–progerin and GST–Smurf2. (h) Proximity ligation assay (PLA) assay indicating sites of direct protein–protein interaction of Flag–lamin A and Myc‐Smurf2 in HEK‐293T cell nuclei (red signal). Cells transfected with Flag–lamin A and empty Myc vector served as a control. Bars, 5 μm. (i) Quantification of lamin A–Smurf2 PLA analysis. ****P <* *.0001. Data are mean ± *SEM* of ten different fields (*n* = 87 cells for Flag–lamin A/Myc‐empty (control); and *n* = 88 cells for Flag–lamin A/Myc‐Smurf2). (j) PLA of the protein–protein interaction of Flag–progerin and Myc‐Smurf2 expressed in HEK‐293T cells. Bars, 5 μm. (k) Quantification of Smurf2–progerin PLA. ****P <* *.0001. Data are mean ± *SEM* of ten different fields (*n* = 88 cells for Flag–Progerin/Myc‐empty (control); and *n* = 97 for Flag–Progerin/Myc‐Smurf2)

Hutchinson‐Gilford progeria syndrome (HGPS) is one of the most severe and devastating laminopathies linked to *LMNA* mutation. Patients with HGPS exhibit characteristics of premature aging and generally die in their teens, mostly due to cardiovascular complications. In most HGPS cases, there is a recurrent heterozygous silent mutation in exon 11 of *LMNA* (c.1824C>T, G608G). This mutation activates a cryptic mRNA splice site that results in the deletion of fifty amino acids near the C‐terminus of prelamin A, yielding a permanently farnesylated and carboxymethylated *dominant* protein named progerin (LAΔ50) (Eriksson et al., 2003; Gonzalo, Kreienkamp & Askjaer, 2017; Figure 1a).

Research aimed at understanding disease progression and treatment options of HGPS led to characterization of many cellular functions for A‐type lamins, revealing their intrinsic role in pathways that are also known to contribute to tumor progression, including chromatin organization and DNA damage response, DNA replication, gene expression, proliferation, and genomic integrity regulation (Taddei, Hediger, Neumann & Gasser, 2004; Gruenbaum, Margalit, Goldman, Shumaker & Wilson, 2005; Lees‐Miller, 2006; Singh et al., 2013; Bell & Lammerding, 2016). These studies also revealed that lamin A coordinates numerous cellular processes and signaling pathways by providing an intranuclear platform for protein‐protein interactions (Dittmer & Misteli, 2011; Kubben, Voncken & Misteli, 2010; Prasad, Kandasamy & Pandey, 2009).

Despite the importance of A‐lamins, the molecular mechanisms implicated in their regulation are elusive. In this study, we identified Smurf2, a HECT type E3 ubiquitin ligase and recently discovered tumor suppressor (Blank et al., 2012; Emanuelli et al., 2017; Zou, Levy‐Cohen & Blank, 2015), as the essential regulator of stability and protein turnover of lamin A and its disease‐associated mutant form progerin. We also determined that protein turnover of A‐lamins is strictly dependent on unaltered catalytic activities of Smurf2 and is executed through the autophagic–lysosomal pathway. Furthermore, we demonstrated that the reciprocal relationship between Smurf2 and A‐lamins is preserved in different types of mouse and human cells and tissues.

## 2 RESULTS

### 2.1 Smurf2 physically interacts with lamin A and progerin

A‐lamins are primarily localized at the nuclear periphery (at the nuclear rim), although they can be also found in the nucleoplasm (Dittmer & Misteli, 2011). Immunohistochemistry (IHC) with IHC‐specific anti‐Smurf2 antibody conducted on a panel of different human tissues revealed that Smurf2, similar to A‐lamins, exhibits nuclear localization, with a noticeable sequestration at the nuclear rim (Figure 1b). Immunofluorescence studies performed in different types of human cells, including primary dermal fibroblasts (HDFs), embryonic kidney HEK‐293T cells, and breast adenocarcinoma MDA‐MB‐231 cells, revealed a co‐localization between lamin A and GFP–Smurf2, in particular at the nuclear envelope (Figure 1c,d, upper panels, and Figure S1a,b). Co‐localization between GFP–Smurf2 and progerin (LA∆50) was monitored in both HEK‐293T cells expressing recombinant Flag–progerin and in patient‐derived HGPS dermal fibroblasts (HGPS HDFs) (Figure 1c,d, bottom panels).

Subsequent co‐immunoprecipitation studies conducted on the endogenous Smurf2 and lamin A in HEK‐293T cells provided further evidence that Smurf2 complexes with lamin A (Figure 1e and Figure S1c).

Next, we conducted in vitro binding assays using purified GST‐Smurf2, Flag–lamin A, and Flag–progerin. The data obtained in these experiments suggested a direct interaction between Smurf2–lamin A and Smurf2–progerin (Figure 1f,g).

Finally, in situ proximity ligation assay (PLA), which enables determining the protein–protein interactions directly within the cell, provided further evidence that Smurf2 interacts with lamin A (Figure 1h,i) and progerin (Figure 1j,k) in cells. Collectively, these data established Smurf2 as a novel binding partner of A‐lamins.

### 2.2 Smurf2 ubiquitinates lamin A and progerin

**Figure 2:**
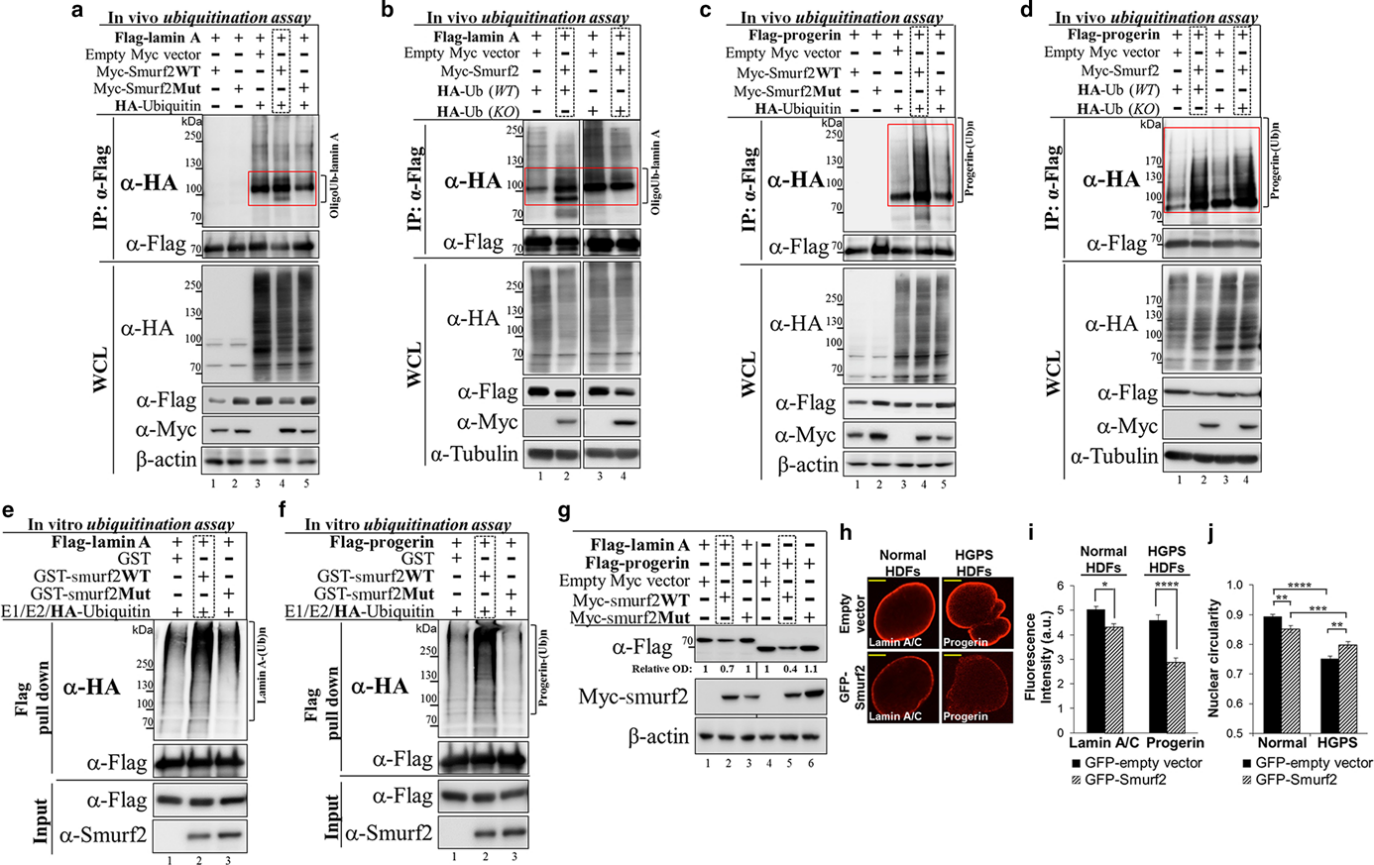
Lamin A and progerin are the targets for Smurf2‐mediated ubiquitination and degradation. (a) Western blot analysis showing that Smurf2 ubiquitinates lamin A in E3 ligase‐dependent manner in vivo (in HEK‐293T cells). This ubiquitination was not detected in cells expressing a catalytically inactive mutant form of Smurf2 (Smurf2Mut). (b) Smurf2 oligo‐ubiquitinates lamin A. Note, the disappearance of lamin A oligo‐ubiquitination in the ubiquitin mutant samples (HA‐UbKO). (c) Western blot analysis showing that Smurf2 ubiquitinates progerin in Smurf2 E3 ligase‐dependent manner. Note, the pattern of Smurf2‐mediated ubiquitination of progerin is different from lamin A. (d) Western blot analyses validating Smurf2‐mediated multi‐ubiquitination of progerin. Rectangles in panels (a–d) indicate the area used for quantification of the effects of Smurf2 on ubiquitination of lamin A and progerin (see Figure S2b–e). (e, f) Western blot analysis of lamin A and progerin ubiquitination by purified recombinant GST‐Smurf2 in the presence of E1, E2, and HA‐tagged Ubiquitin (in vitro ubiquitination assay). Smurf2 mutant protein and GST were used as controls. Flag–lamin A and Flag–progerin were pulled down from the reaction using FLAG antibody. (g) Western blot analysis showing that Smurf2 decreases cellular levels of both lamin A and progerin expressed in HEK‐293T cells in E3 ligase‐dependent manner. (h) Confocal images showing effects of Smurf2 overexpression (GFP–Smurf2) on cellular levels of lamin A and progerin in normal and progeria fibroblasts. Cells expressing an empty GFP vector served as a control. Bars, 5 μm. (i) Quantification of the confocal data shown in panel (h). *P <* *.05 and ****P <* *.0001. Data are mean ± *SEM* of three independent experiments (*n* = 255 cells/group). (j) Analysis of nuclear circularity in normal and HGPS HDFs transduced with either an empty GFP vector or with GFP–Smurf2. Data are mean ± *SEM*. At average, 66 normal HDFs (*n* = 2) and 101 HGPS cells (*n* = 3) were analyzed per group. **P <* *.01, ***P <* *.001, and ****P <* *.0001

Smurf2 is a HECT type E3 ubiquitin ligase that can directly ubiquitinate its binding partners/substrates. Active‐site cysteine of Smurf2 (Cys716), which makes a transient thioester bond with ubiquitin, is crucial for Smurf2 catalytic activity (Kavsak et al., 2000; Zhang, Chang, Gehling, Hemmati‐Brivanlou & Derynck, 2001). To examine whether lamin A and progerin are the substrates for Smurf2‐mediated ubiquitination, we conducted several lines of experiments. First, we demonstrated that Smurf2 is capable of ubiquitinating lamin A in cells (in vivo ubiquitination assay), yielding oligo‐ubiquitination of lamin A (Figure 2a,b and Figure S2a–c). Oligo‐ubiquitinated lamin A was discernible as distinct bands of ubiquitin (Ub)‐conjugated lamin A migrating in SDS‐PAGE at ~90–115 kDa (Figure 2a,b, lanes 4 and 2, respectively; and Figure S2a, lane 2). This ubiquitination was strictly dependent on unaltered E3 ubiquitin ligase function of Smurf2, as Smurf2's catalytically inactive mutant (Cys716Gly; Smurf2Mut) failed to produce this phenomenon (Figure 2a, lane 5 vs. 4; Figure S2a, lane 3 vs. 2; and Figure S2b). Of note, the Smurf2‐mediated ubiquitination of lamin A, and its dependence on Smurf2 enzymatic activities, was also observed under very harsh lysis conditions: when cells instead of a RIPA buffer were lysed in 1% SDS followed by immediate sample boiling and sonication (not shown).

The oligo‐ubiquitination of lamin A was further validated using ubiquitin mutant (HA‐UbKO) vs. ubiquitin wild‐type form (HA‐UbWT). In HA‐UbKO mutant form, all the lysines (seven in total) were mutated to arginines, abolishing the ability of ubiquitin molecules to form oligo/poly‐ubiquitin chains. The data show that the substitution of wild‐type ubiquitin with its mutant abolished Smurf2‐mediated oligo‐ubiquitination of lamin A (Figure 2b, lane 4 vs. 2; and Figure S2c).

Lamin A mutant form progerin (LA∆50) was also found to be ubiquitinated by Smurf2 in E3 ligase‐dependent manner (Figure 2c, lane 4; and Figure S2d). However, the data suggested that in contrast to lamin A, progerin was apparently multi‐ubiquitinated by Smurf2: ubiquitinated‐progerin migrated in SDS‐PAGE at multiple sites ranging from ~80 to 250 kDa. Subsequent studies incorporating wild‐type vs. mutant form of ubiquitin provided further evidence that Smurf2 mediates multi‐ubiquitination of progerin: Ubiquitination pattern of progerin remained unaltered in HA‐UbKO‐expressing cells as compared to HA‐UbWT‐expressing cells (Figure 2d, lane 4 vs. 2; and Figure S2e).

In a follow‐up in vivo ubiquitination study, incorporating the affinity purification of lamin A and progerin coupled with mass spectrometry analysis, we validated the dependency of Smurf2‐mediated ubiquitination of lamin A and progerin on unaltered enzymatic activities of Smurf2. These findings will be published elsewhere.

Next, using an ubiquitination reconstitution assay (in vitro ubiquitination assay) incorporating purified ubiquitin‐activating enzyme (E1), ubiquitin conjugase (E2), HA‐ubiquitin, GST‐Smurf2 (either wild‐type or E3 ligase‐mutant form), and Flag–lamin A or Flag–progerin, we showed that Smurf2 is capable to directly ubiquitinate lamin A and progerin in E3 ligase‐dependent manner (Figure 2e,f and Figure S2g). Of note, to ensure that Smurf2‐mediated ubiquitination of lamin A and progerin belongs to these proteins, and not to Smurf2 (e.g., due to its possible auto‐ubiquitination) or any other components used in the ubiquitination reaction, we conducted a set of validation studies, which confirmed the specific formation of ubiquitin conjugates on A‐lamins (Figure S2f,h).

### 2.3 Smurf2 negatively regulates levels of lamin A and progerin

To investigate the consequences of Smurf2‐mediated ubiquitination of lamin A and progerin, we performed the following sets of experiments. Firstly, we expressed these proteins in HEK‐293T cells together with either Smurf2 wild‐type (Smurf2WT) or its E3 ligase‐deficient mutant (Smurf2Mut) and analyzed the steady‐state levels of lamin A and progerin in these cells. The data (Figure 2g and Figure S2i) show reduced levels of lamin A and progerin when co‐expressed with wild‐type form of Smurf2. Consistent with the previous observations, Smurf2 catalytically inactive mutant failed to decrease levels of lamin A and progerin. Furthermore, the results indicated that effect of Smurf2 on A‐lamins was proportional to the amount of Smurf2 transduced to the cells (Figure S2j).

Subsequently, we analyzed the effects of Smurf2 overexpression on cellular levels of lamin A and progerin in primary human dermal fibroblasts (HDFs) derived either from a healthy individual or from patient with HGPS, using a confocal microscopy analysis. The results revealed that similar to HEK‐293T cells, overexpression of Smurf2 in HDF cells significantly decreased cellular levels of A‐lamins, in particular of progerin (Figure 2h,i).

Furthermore, we measured the nuclear circularity in HDF normal and HGPS cells transduced with GFP–Smurf2 or an empty GFP vector (control). In accordance with the previously published studies (Goldman et al., 2004; Scaffidi & Misteli, 2005), the nuclear circularity in HGPS cells was significantly decreased as compared to normal cells (Figure 2j). Remarkably, overexpression of Smurf2 in progeria fibroblasts was able to significantly reduce the nuclear deformability and improve the nuclear circularity in patient cells. Of note, in normal HDFs the nuclear circularity was decreased (Figure 2j).

### 2.4 Inactivation of Smurf2 either through RNAi or genomic ablation increased the steady‐state levels of lamin A in cells and tissues

**Figure 3:**
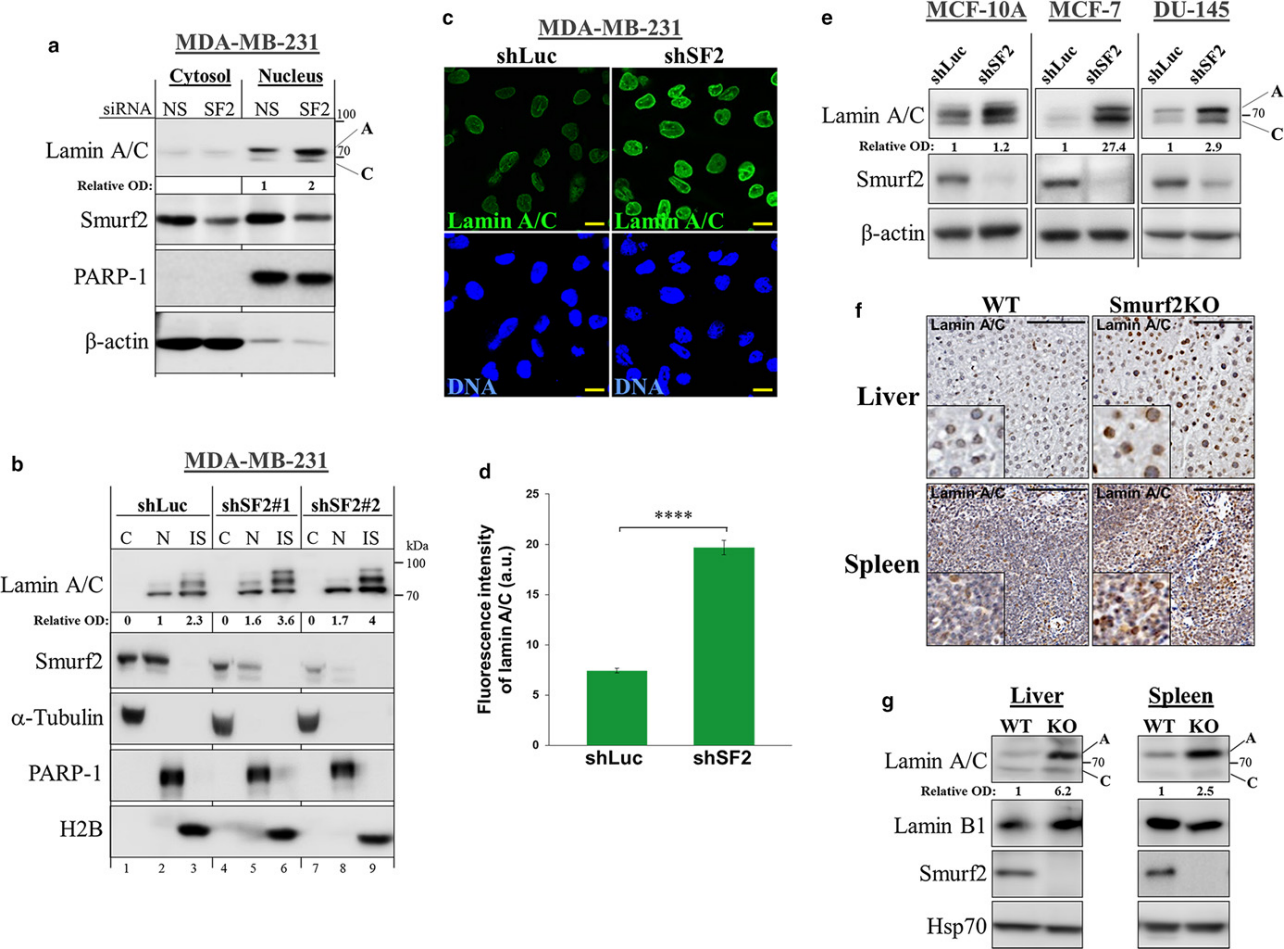
Smurf2 depletion increases the steady‐state levels of lamin A in cells and tissues. (a) Western blot analysis showing increased cellular levels of lamin A after siRNA‐mediated knockdown of Smurf2 (SF2) in MDA‐MB‐231 cells. NS (nonspecific siRNA) used as a control. (b) Western blot analysis showing the effect of Smurf2 knockdown with lentiviral‐based shRNAs on lamin A. Analyses were performed on fractionated samples in which cytosolic (C), nucleoplasmic (N), and insoluble cellular fractions (IS) were extracted. Proteins associated with insoluble fraction were extracted using sonication. Protein loadings and degree of fractionations are demonstrated using antibodies against the cytosolic α‐tubulin, the nucleoplasmic PARP‐1, and a component of the insoluble fraction–histone H2B. Note, the increase in lamin A in Smurf2 knockdown cells was proportional to the efficiency of Smurf2 depletion. In the insoluble fractions, lamin A appears in three distinct forms. (c) Immunofluorescence staining of lamin A/C in MDA‐MB‐231 cells showing increased lamin A/C levels upon Smurf2 knockdown. DNA was counterstained with Hoechst 33258. Bars, 10 μm. (d) Quantification of the immunofluorescent data obtained on Smurf2 knockdown MDA‐MB‐231 cells. ****P <* *.0001. Data are mean ± *SEM* of ten different fields (*n* = 69 cells/group). (e) Western blot analysis of lamin A/C levels in different human cell models knocked down for Smurf2. (f) IHC staining of lamin A/C (brown) in liver and spleen tissue sections prepared from WT and Smurf2 knockout mice. The nuclei were counterstained with hematoxylin (blue). Bars, 100 μm. Both WT and KO tissues were sampled on the same slide and processed for IHC simultaneously. The images were acquired and processed under the equal settings. (g) Western blot analysis of lamin A/C and lamin B1 expression in liver and spleen tissues of WT and Smurf2KO mice. Note, lamin A was a predominant isoform increased in Smurf2‐depleted mouse tissues

Next, we examined the effect of Smurf2 knockdown on the steady‐state levels of A‐lamins. To this end, we transfected human breast adenocarcinoma MDA‐MB‐231 cells with either nonspecific siRNA (siNS) or with siSmurf2 (siSF2), and examined protein levels of A‐lamins 72 hr after transfection. The data (Figure 3a and Figure S3a) showed that the steady‐state levels of lamin A were prominently increased after the Smurf2 knockdown. Similar results were also obtained when Smurf2 was knocked down with lentiviral‐based shRNAs (Figure 3b). These effects were monitored through the use of two different shRNAs designed to target Smurf2 at either its coding sequence (shSF2#1) or 3′UTR (shSF2#2), and were proportional to the efficiency of Smurf2 knockdown (Figure 3b, lanes 7–9 vs. 4–6 and 1–3). Subsequent immunofluorescent studies conducted on shSF2 cells showed significantly increased levels of A‐lamins also at a single‐cell level (Figure 3c,d). Real‐time qRT–PCR analyses of lamin A/C mRNA expression in these cells pointed to post‐transcriptional level of regulation of A‐lamins by Smurf2 (Figure S3b).

The increased levels of A‐lamins after Smurf2 knockdown were also observed in other human cell models: in mammary epithelial MCF‐10A cells, breast adenocarcinoma MCF‐7 cells, and in prostate carcinoma DU‐145 cells. In all these cells, decrease in Smurf2 expression through shRNA increased the steady‐state levels of A‐lamins (Figure 3e).

Immunohistochemistry and Western blot analyses conducted on the tissue samples of Smurf2‐deficient vs. littermate control mice revealed that increased protein levels of lamin A were also a characteristic of Smurf2‐ablated tissues (Figure 3f,g and Figure S3c,d). Altogether, these data indicated that Smurf2 is a key regulator of the steady‐state levels of A‐lamins.

### 2.5 Smurf2 regulates stability and protein turnover of A‐lamins through the autophagic–lysosomal degradation pathway

**Figure 4:**
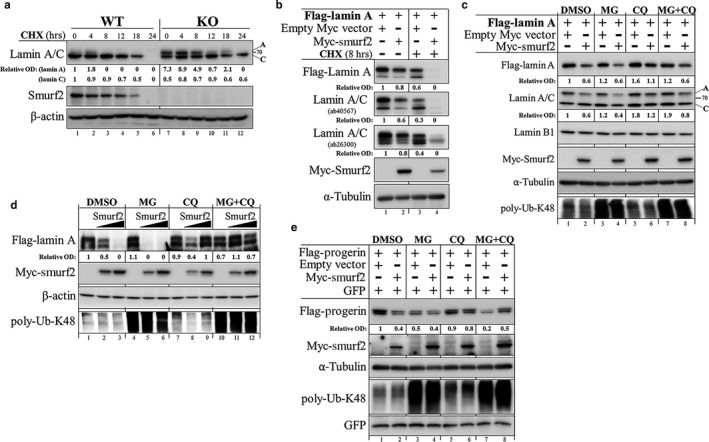
Smurf2 regulates the stability of lamin A and progerin through the lysosomal proteolysis. (a) Western blot analysis of lamin A/C levels and protein turnover in Smurf2KO MEFs. CHX—cycloheximide. (b) Western blot analysis showing the effects of Smurf2 on the levels of Flag–lamin A and endogenous lamin A/C in CHX‐treated and untreated HEK‐293T cells. The decrease in lamin A/C in Smurf2 overexpressing cells was detected using two different antibodies for lamin A/C, recognizing these proteins at two distinct epitopes. (c) Western blot analysis showing that Smurf2‐mediated degradation of lamin A can be rescued by the inhibition of the lysosomal degradation pathway. HEK‐293T cells were co‐transfected with Flag–lamin A and Myc‐Smurf2, or with an empty Myc vector. Twenty‐four hours later, cells were treated with either proteasome inhibitor MG132 (MG; 2.5 μm), lysosomal inhibitor chloroquine (CQ; 20 μm), or with a combination of both for additional 4 hr. Cells were then lysed in RIPA buffer, and cell extracts analyzed in Western blot with the indicated antibodies. Inhibition of the proteasomal degradation pathway was verified with anti‐poly‐ubiquitin‐Lys48 (poly‐Ub‐K48) antibody. Note that Smurf2 predominantly affected the stability of lamin A. (d) Repetition of the experiments described in (c) with gradually increased concentrations of Myc‐Smurf2 (0; 2; 4 μg), and sample sonication. (e) Western blot analysis showing that Smurf2‐mediated degradation of progerin can be rescued by the inhibition of the lysosomal breakdown pathway

To investigate how Smurf2 regulates protein levels of A‐lamins, we conducted several sets of experiments. First, we analyzed the turnover rate of endogenous lamin A/C proteins in Smurf2‐ablated MEF cells. We found that cellular levels of lamin A were significantly increased in Smurf2 knockout MEFs (Figure 4a, lane 7 vs. 1), and had a slower turnover after blocking protein synthesis with cycloheximide (Figure 4a, lanes 8–11 vs. 2–5). Consistently, overexpression of Smurf2 accelerated protein turnover of both endogenous and recombinant Flag–lamin A expressed in HEK‐293T cells, in particular after the cycloheximide treatment (Figure 4b and Figure S3e).

Next, we analyzed the Smurf2‐mediated degradation of lamin A following cell treatment with the proteasomal inhibitor MG‐132 and/or autophagic/lysosomal inhibitor chloroquine (CQ). Consistent with our previous observations, adding Smurf2 to the cells led to a prominent reduction in cellular levels of lamin A (Figure 4c). The data also revealed that Smurf2‐mediated proteolysis of lamin A could be rescued through the inhibition of lysosomal but not proteasomal protein degradation (Figure 4c, lane 6 vs. 4 and 2). To validate these observations, we conducted this protein degradation assay again, but at this time we expressed Smurf2 in gradually increasing concentrations. The data (Figure 4d) showed that Smurf2‐mediated reduction in lamin A levels is proportional to the amount of Smurf2 transduced to the cells and that lamin A protein turnover occurs through the autophagic–lysosomal pathway. Similar results were also obtained with progerin (Figure 4e). Again, only the inhibition of the autophagic/lysosomal degradation pathway with chloroquine rescued progerin from the Smurf2‐mediated degradation (Figure 4e, lane 6 vs. 4 and 2).

**Figure 5:**
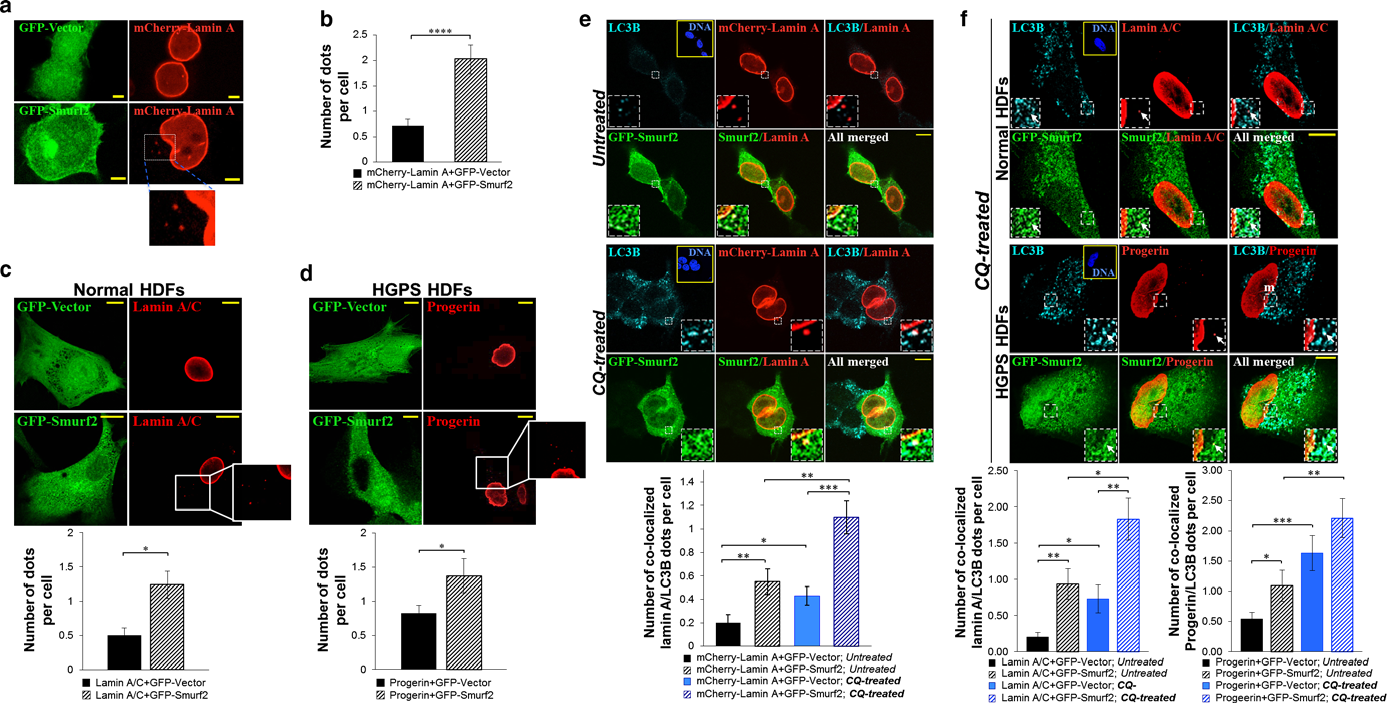
Smurf2‐mediated turnover of lamin A and progerin involves the autophagic–lysosomal pathway. (a) Confocal images showing that co‐expression of GFP–Smurf2 and mCherry‐lamin A in HEK‐293T cells increases the number of mCherry‐lamin A's cytoplasmic dots. Cells co‐expressing mCherry‐lamin A and an empty GFP vector served as a control. Bars, 5 μm. (b) Quantification of the confocal data shown in (a). ****P <* *.0001 using Student's *t*‐test. Data are mean ± *SEM* of three independent experiments (*n* = 135 cells/group). (c, d) Confocal images showing that overexpression of GFP–Smurf2 in human normal and HGPS fibroblasts increases the lamin A and progerin cytoplasmic dots. Bars, 10 μm. Quantification of the data is shown on the bottom of these panels. *P <* *.05. Data are mean ± *SEM* of three independent experiments with an average of 100 cells/group for normal HDFs and 114 cells/group for HGPS cells. (e) Confocal images of untreated and chloroquine‐treated HEK‐293T cells co‐expressing GFP–Smurf2 and mCherry‐lamin A, and immunostained with anti‐LC3B antibody (autophagosome marker). Bars, 10 μm. Quantification of the confocal data is shown on the bottom. *P <* *.05, **P <* *.01, ***P <* *.001. Data are mean ± *SEM* of two independent experiments (*n* = 89 cells/group). (f) Confocal images showing co‐localization of LC3B with lamin A and progerin in normal and HGPS HDFs expressing GFP–Smurf2. Arrows point to co‐localization sites of all three proteins in the cytoplasmic compartment of cells. Bars, 10 μm. Quantification of the data is shown on the bottom. At average 44–50, GFP‐positive cells were analyzed and quantified in each group. Data are mean ± *SEM*

To corroborate our biochemical data on Smurf2‐mediated breakdown of A‐lamins via the autophagic–lysosomal pathway, we conducted the following experiments. First, using a direct immunofluorescent confocal analysis of HEK‐293T cells expressing mCherry‐lamin A together with the GFP–Smurf2 (or an empty GFP vector as a control), we demonstrated that in Smurf2 overexpressing cells the number of mCherry‐lamin A's cytoplasmic dots was significantly increased (Figure 5a,b). Remarkably, a similar phenomenon was also observed in primary HDFs derived either from a healthy individual or from patient with HGPS (Figure 5c,d). Second, we repeated these experiments again, but now incorporated into the experimental analysis also immunostaining with the anti‐LC3B antibody. The analyses were conducted on chloroquine‐treated and untreated cells. LC3B is a central protein in the autophagic–lysosomal protein degradation pathway and is used to monitor lysosomal activity (Barth, Glick & Macleod, 2010; Shintani & Klionsky, 2004). For the immunostaining, we used anti‐LC3B antibody from Cell Signaling (Cat. #2775). This antibody has a much stronger reactivity for the endogenous type II form of LC3B (LC3B‐II), which is specifically localized to autophagic structures throughout the process from phagophore to lysosomal degradation. LC3B‐II has been documented to accumulate in the autophagic–lysosomal compartment of the cell following chloroquine treatment. Using this experimental setup, we confirmed that Smurf2‐mediated degradation of both lamin A and progerin occurs through autophagy, as evident by co‐localization of lamin A and progerin cytoplasmic dots with LC3B (Figure 5e,f), and with the lysosomal‐associated membrane protein LAMP1 (Figure S4).

### 2.6 The Smurf2–lamin A relationship is preserved in human normal and cancer tissues

To determine the relationship between Smurf2 and A‐type lamins in human tissues, we obtained a collection of human normal and cancer tissues (tissue microarrays—TMAs) from US Biomax and stained these tissues with IHC‐specific anti‐Smurf2 and antilamin A/C antibodies. The following TMAs were stained and analyzed: (i) multi‐organ normal TMA (FDA999 m; *N* = 96), containing 32 types of human normal organs/tissues taken from three normal individuals; (ii) breast cancer TMA with matched adjacent normal breast tissue (BR804a; *N* = 76); (iii) breast invasive ductal carcinoma with matched metastatic carcinoma tissue (BR10010c; *N* = 98); and (iv) prostate cancer TMA containing both tumor and normal prostate tissues (PR1921; *N* = 192). We chose breast and prostate tumor TMAs based on our results demonstrating that Smurf2 knockdown in these cells increased the expression levels of A‐lamins (Figure 3a–e). Histopathological evaluation of TMAs was conducted by two independent board‐certified pathologists. The staining intensity and percentages of positive cells were scored using the standard scoring system: 0 = <10%; 1 = 10%–24%; 2 = 25%–49%; 3 = 50%–74%; 4 = 75%–100%.

**Figure 6:**
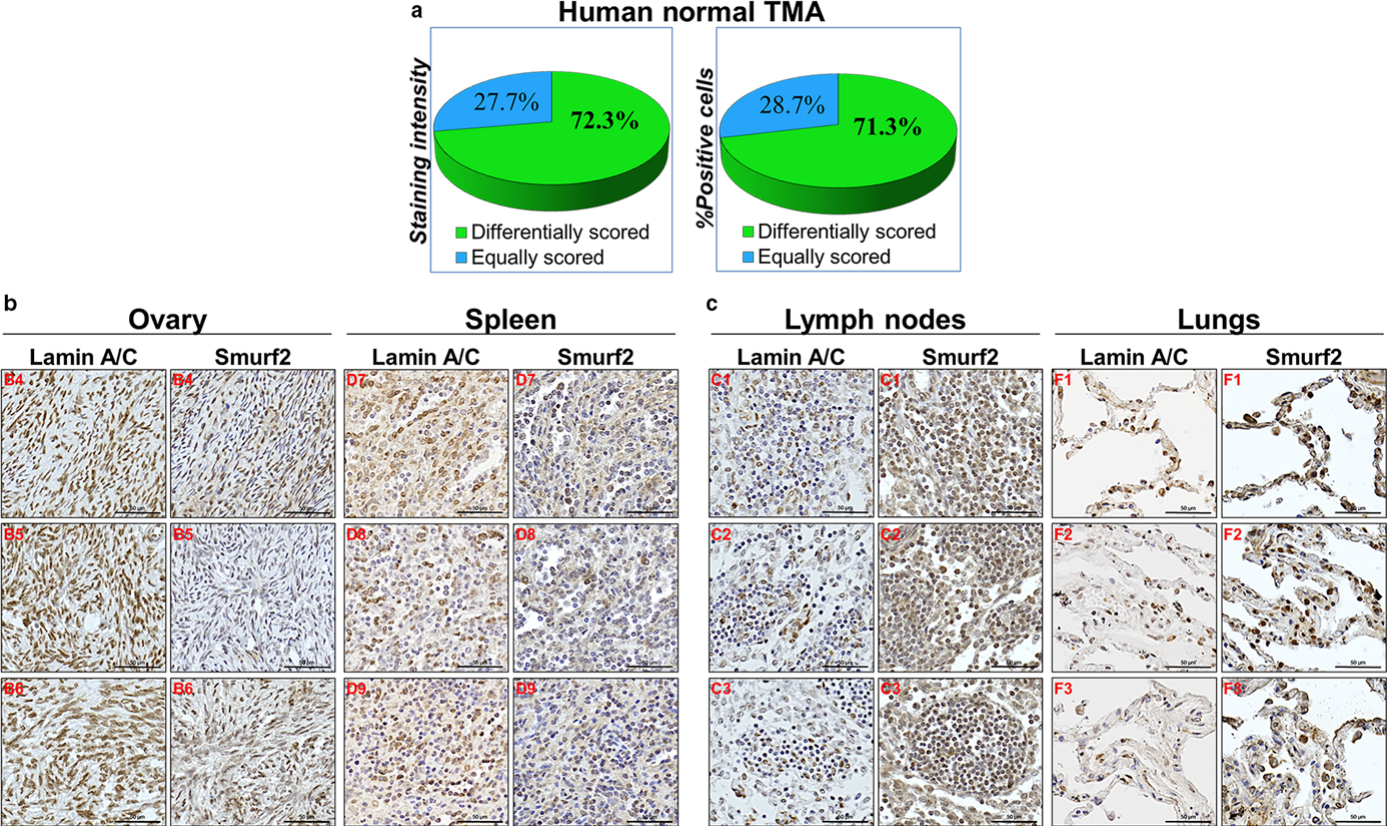
The Smurf2–lamin A relationship is preserved in human normal tissues. (a) Summary of the quantification analysis of Smurf2 and lamin A/C intensity and percentage of positive cells in a panel of human normal tissues. Smurf2 and lamin A IHC staining was conducted on sequentially sectioned TMAs. (b) Representative images of IHC‐stained tissues of three normal individuals showing high levels of lamin A/C and lower levels of Smurf2. B4, B5, B6, and D7, D8, D9 are the coordinates of the samples in the tissue array. Bars, 50 μm. (c) Representative images of tissues showing high levels of Smurf2 and lower levels of lamin A/C. C1, C2, C3, and F1, F2, F3 are the coordinates of the samples in the tissue array. Bars, 50 μm

Analysis of normal tissue samples (Figure 6a) revealed that in more than 70% of samples Smurf2 and A‐lamins were differentially scored: Samples with high expression levels of Smurf2 showed lower expression of A‐lamins; and *vice versa*: Samples with low expression of Smurf2 showed higher expression levels of lamin A/C. The most prominent differences between the expression levels of Smurf2 and A‐lamins were observed in: (i) ovary, spleen, thyroid gland, muscle, and endometrium tissues. In all these tissues, we detected high levels of lamin A/C and low levels of Smurf2 (Figure 6b and Figure S5a); (ii) in contrast, high levels of Smurf2 and lower levels of A‐lamins were observed in lymph node, lungs, liver, small intestine, and pancreatic tissues (Figure 6c and Figure S5b). We did not detect consistent differences between expression of Smurf2 and lamin A/C in brain, colon, kidney, and skin. These findings suggest that Smurf2 affects A‐lamins in a tissue‐specific manner.

We have also observed the reciprocal relationship between decreased levels of A‐lamins and increased levels of Smurf2, and *vice versa*, in cancer tissues. The percentage of samples with differential Smurf2–lamin A/C scores in cancer TMAs ranged between approximately 40% and 80%, dependent on the tumor type (Figures S6 and S7).

## 3 DISCUSSION

In this study, we identified a previously unknown guiding arm in the regulation of expression of A‐lamins in mammalian cells and tissues. We found that Smurf2, a HECT type E3 ubiquitin ligase and recently suggested tumor suppressor, functions as a physiological regulator of A‐lamins, in particular of lamin A and its disease‐associated mutant form progerin, fine‐tuning their expression levels. We showed that Smurf2 operates as a *bona fide* E3 ubiquitin ligase that directly binds, ubiquitinates, and negatively regulates the expression levels of lamin A and progerin in Smurf2 dose‐ and E3 ligase‐dependent manners. Consistently, we demonstrated that overexpression of catalytically active Smurf2 promoted the autophagic–lysosomal breakdown of A‐lamins, whereas the depletion of Smurf2 significantly increased the lamin A levels. These results were observed in Smurf2 genetically ablated mouse cells and tissues, as well as in Smurf2 knockdown human cells. Our study also incorporated the usage of HDF cells derived from a healthy individual or from patient with HGPS. Analyses conducted in these cells provided further support for the role of Smurf2 in the regulation of A‐lamins. Remarkably, the overexpression of Smurf2 in progeria patient fibroblasts significantly reduced the nuclear deformability in these cells. In normal HDFs, the nuclear circularity was decreased upon Smurf2 overexpression, consistent with our other data showing that Smurf2 is an essential negative regulator of both progerin and lamin A.

Finally, the data obtained in IHC studies of tissues from Smurf2‐deficient and wild‐type mice, as well as on tissue microarrays incorporating more than 460 human normal and cancer tissues, provided additional support for Smurf2 as a regulator of A‐lamins. Taken as a whole, the evidence supports the model whereby Smurf2 acts as a physiological regulator of A‐lamins.

A few recent studies reported that A‐lamins undergo protein degradation under different conditions. AKT1‐mediated phosphorylation and subsequent degradation of lamin A/C were observed in epidermal keratinocytes during terminal differentiation (Naeem, Zhu, Di, Marmiroli & O'Shaughnessy, 2015). In another study, overexpression of AIMP3/p18, which is normally associated with the macromolecular tRNA synthetase complex, was shown to accelerate cellular senescence and promote proteasome‐mediated degradation of lamin A, but not of lamin C, prelamin A, or progerin (Oh et al., 2010). Our data showed that under normal growth conditions the turnover of both lamin A and progerin occurred through the autophagic–lysosomal breakdown (Figures 4 and 5, Figure S4). Intriguingly, while cell treatment with the proteasome inhibitor MG‐132 had no obvious effect on lamin A (Figure 4c, lane 3 vs. 1), this treatment decreased the progerin levels (Figure 4e, lane 3 vs. 1). In line with this finding, a recent study demonstrated that MG‐132 could promote the degradation of progerin by activation of autophagy (Harhouri et al., 2017).

The current study established Smurf2 as an E3 ubiquitin ligase that regulates cellular levels of both progerin and lamin A through the autophagic–lysosomal degradation. Interestingly, the data also indicated that in mouse tissues and human HEK‐293T cells, Smurf2 had a little, if any, the effect on lamin C or lamin B1 (Figure 3g and Figure S3c), implying the specificity of Smurf2 in the regulation of A‐lamins.

A‐lamins, in particular progerin, have been associated with a physiological aging, in addition to HGPS. There is evidence that progerin progressively accumulates in different tissues of normal individuals during their aging (Butin‐Israeli, Adam, Goldman & Goldman, 2012). Similar to A‐lamins, Smurf2 also appears to be associated with aging and age‐related diseases, including cancer. Recently, we and others showed that under the stress of aging mice knockout for Smurf2 develop a wide spectrum of tumors in different organs and tissues (Blank et al., 2012; Ramkumar et al., 2012; Zou et al., 2015). Moreover, increased cycling and reduced quiescence of hematopoietic stem cells in Smurf2‐deficient aging mice was also reported (Ramkumar, Kong, Trabucco, Gerstein & Zhang, 2014).

In another study, Wu et al. demonstrated that overexpression of Smurf2 led to accelerated cartilage degradation and that ectopic expression of Smurf2 also facilitated the degeneration of the lumbar intervertebral disk, both of which are age‐related processes (Wu & Huang, 2017; Wu et al., 2008). Accordingly, Smurf2‐deficient mice were less susceptible to the pathological development of osteoarthritis compared to wild‐type animals (Huang, Veien, Zhang, Ayers & Song, 2016).

More intriguing results were recently published by Mayeux's group. The authors analyzed the data from a cohort of families with exceptional longevity and healthy aging (4,289 individuals) and found that Smurf2 was significantly associated with the leukocyte telomere length at gene‐wise level (Lee et al., 2014).

Taken together, these findings strongly suggest that Smurf2, similar to A‐lamins, is intrinsically involved in aging and age‐related diseases, including cancer. Interestingly, both the *SMURF2* and *LMNA* genes are not frequently altered in human malignancies; however, changes in the expression of these genes are common in many cancers (Bell & Lammerding, 2016; Zou et al., 2015). Moreover, the expression pattern of these proteins in tumors could be highly heterogeneous (Figures S6 and S7). Despite the heterogenic expression of Smurf2 and A‐lamins, our results pointed to the reciprocal relationship between these proteins also in human cancer tissues. This observation is particularly intriguing because identification of the role of the Smurf2–lamin A axis in cancer may lead to the development of potential screening techniques that can direct appropriate therapies to the patients based on molecular profiling of the biopsy tissue. Another ramification of this study is that our findings on Smurf2‐mediated proteolysis of progerin lay a foundation for evaluating the efficiency of progerin clearance by Smurf2 as a possible therapeutic approach in progeria treatment.

## 4 EXPERIMENTAL PROCEDURE

### 4.1 Cell cultures

Human dermal fibroblasts (HGADFN167, HGFDFN168) were obtained from the Progeria Research Foundation. Smurf2 knockout (*Smurf2* ^−/−^) and wild‐type MEF cells were generously provided by Dr. Ying Zhang (NIH). Cells were maintained in high‐glucose DMEM (GIBCO) supplemented with l‐glutamine, fetal bovine serum (15% for HDFs, and 10% for MEFs and human cancer cell strains), and 1% (v/v) Pen‐Strep (GIBCO). Human mammary epithelial MCF10A cells were cultured in DMEM/F12 supplemented with l‐glutamine, 5% donor horse serum, 20 ng/ml epidermal growth factor, 10 μg/ml insulin (Biological Industries), 0.5 μg/ml hydrocortisone, 100 ng/ml cholera toxin (Sigma), and 1% (v/v) Pen‐Strep. All cell cultures were maintained at 37°C with 5% CO_2_.

### 4.2 Animals

C57BL/B6 Smurf2 knockout and control wild‐type mice were housed at the Faculty SPF animal facility according to Federation of Laboratory Animal Science Associations (FELASA) guidelines. All experimental protocols were approved by the Animal Care and Use Committee of Bar‐Ilan University.

### 4.3 Protein extraction and Western Blot

For the preparation of whole cell lysates (WCL), cells were re‐suspended in RIPA buffer (50 mm Tris‐HCl [pH 7.8], 1% Nonidet P40 Substitute (NP‐40 buffer; Sigma), 150 mm NaCl, 0.1% SDS, 0.5% sodium deoxycholate) supplemented with protease (Roche) and phosphatase inhibitors (Sigma). Samples were maintained on ice for 30 min and then sonicated for 1 min at 30% amplitude. Fractionated samples were prepared as previously described (Blank, Lerenthal, Mittelman & Shiloh, 2006).

For protein extraction from mouse tissues, the collected organs were homogenized in RIPA buffer using TissueRuptor (Qiagen) and, following sonication, cleared using centrifugation. Protein concentrations were assessed using Pierce BCA protein assay kit (Thermo Fisher Scientific). Samples were analyzed by Western blot analysis using the following antibodies: antilamin A/C (ab26300, 1:1,000 and ab40567, 1:500; Abcam), antilamin B1 (#12586, 1:1,000; Cell Signaling), anti‐PARP1 (#9532, 1:5,000; Cell Signaling), anti‐β‐actin (#600401886, 1:2,000; Rockland), anti‐α‐tubulin (T9026, 1:2,000; Sigma), anti‐H2B (ab1790, 1:1,000; Abcam), anti‐Hsp70 (ab45133, 1:10,000; Abcam), anti‐GFP (11814460001, 1:1,000, Roche), anti‐Smurf2 (#12024, 1:1,000; Cell Signaling), anti‐MYC (#2278, 1:1,000; Cell Signaling), anti‐FLAG (F3165, 1:2,000; Sigma), anti‐HA (715500, 1:250; Invitrogen), and anti‐poly‐Ub‐Lys48 (#8081, 1:1,000; Cell Signaling). Horseradish peroxidase‐conjugated secondary antibodies (Jackson Laboratories) were used at the dilution of 1:10,000. The membranes were visualized in the SyngeneG:BOX. Quantification of the data obtained in Western blot analysis was conducted using Gel.Quant.NET.

### 4.4 Immunoprecipitation

For co‐immunoprecipitation (co‐IP) experiments, cells were lysed using different lysis conditions: 0.5% NP‐40 buffer (25 mm Tris‐HCL [pH 7.5], 137 mm NaCl, 1 mm EDTA, 1 mm EGTA, 5% glycerol); 1%‐NP‐40 buffer (1% NP‐40 substitute, 25 mm Tris‐HCL [pH 7.5], 137 mm NaCl, 1 mm EDTA, 1 mm EGTA, 5% glycerol); a freeze‐thawing (FT) buffer (600 mm KCl, 20 mm Tris‐HCl [pH 7.8], 20% glycerol, protease and phosphatase inhibitors), followed by re‐suspension buffer (45 mm Tris‐HCl [pH 7.8], 2.25 mm EDTA, 0.1% NP‐40 buffer); or RIPA buffer. Lysates derived from the same samples were incubated overnight at 4°C with anti‐Smurf2 antibody (sc‐25511; Santa Cruz) or rabbit IgG as a control (I5006; Sigma). Protein G–Sepharose beads (4 Fast Flow; GE Healthcare) were then added, and the samples were incubated for additional 2 hr at 4°C under rotation. Subsequently, beads were washed four times with an ice‐cold lysis buffer and boiled for 5 min in 5× SDS loading buffer (50 mm Tris‐HCl [pH 8], 5 mm EDTA, 5% SDS, 50% glycerol, 50 mm DTT, 0.05% p/v bromophenol blue, 6% 2‐mercaptoethanol).

### 4.5 Immunofluorescence and confocal analysis

Cells were cultured overnight on poly‐d‐lysine covered glass slides and fixed in 4% formaldehyde for 20 min. Then, cells were permeabilized with 0.5% Triton X‐100 in PBS, blocked in 3% BSA, and stained for 1 hr at room temperature with antibody against lamin A/C (Abcam). Subsequently, cells were washed and incubated for 1 hr with goat anti‐rabbit or goat anti‐mouse secondary antibodies conjugated with Alexa‐Fluor 488 (711‐546‐152) or Rhodamine Red™‐X (115‐296‐071), both from Jackson Laboratories. DNA was counterstained with Hoechst 33258 (B2883; Sigma), and cells were analyzed using a LSM780 Inverted Confocal Microscope (Zeiss) through a Plan‐Apochromat 63×/1.40 Oil DIC M27 objective.

For LC3B and LAMP1 staining experiments, HEK‐293T cells were co‐transfected with mCherry‐lamin A and GFP–Smurf2 or with mCherry‐lamin A and GFP‐empty vector, as a control. Sixteen hours later, cells were seeded on coverslips precoated with poly‐d‐lysine, allowed to adhere, and then treated with chloroquine overnight (50 μm for HEK‐293T cells; and 20 μm for HDF cells). Immunostaining was conducted using anti‐LC3B antibody (#2775, 1:100; Cell Signaling) or anti‐LAMP1 antibody (ab24170, 1:100, Abcam), and fluorophore‐labeled secondary antibody (711‐606‐152; Jackson Laboratories). All comparative images were obtained under identical microscope and camera settings and quantified using ImageJ (NIH) software.

### 4.6 Proximity ligation assay

For PLA assay, HEK‐293T cells were transfected with Flag–lamin A (or Flag–progerin) and Myc‐Smurf2. Cells co‐transfected with Flag–lamin A (or progerin) together with empty Myc vector served as controls. PLA assays were performed using Duolink In Situ Red Starter Kit Mouse/Rabbit (DUO92101, Sigma) and anti‐Flag (F3165, 1:1,000; Sigma)/anti‐Myc (#2278 1:100; Cell Signaling) antibodies, according to the manufacturer's instructions.

### 4.7 Tissue microarrays (TMAs) and Immunohistochemistry

Human tissue microarrays (TMAs) were purchased from US Biomax, Inc (Rockville, MD). Mice tissues were fixed in 4% PFA, and 5‐μm tissue sections were prepared. IHC was conducted using anti‐lamin A/C antibody (#2032, 1:100; Cell Signaling) and anti‐Smurf2 (sc‐25511 (H‐50), 1:50; Santa Cruz Biotechnology), as previously described (Blank et al., 2012; Emanuelli et al., 2017). All comparable samples were sampled on the same slide, and all staining procedures were conducted on slides positioned horizontally.

## CONFLICT OF INTEREST

The authors declare no potential conflict of interests.

## AUTHORS’ CONTRIBUTION

APB performed the majority of the experiments and participated in data analysis. AE conducted IHC studies; PAS and NI performed PLA assays, as well as conducted a part of the immunofluorescent studies. In addition, AE, PAS, and NI performed all experiments and data analysis during the manuscript revision. LAP and BP analyzed and scored the TMAs. DMA and GLC performed mouse colony handling, genotyping, and protein extractions from mouse tissues. PK performed co‐IP studies. MB conceived the study, supervised the project, and wrote the manuscript.
