# Supplementary material for: Smurf2 regulates stability and the autophagic–lysosomal turnover of lamin A and its disease‐associated form progerin
Source: Aging Cell. 2018 Feb 5;17(2):e12732. doi: 10.1111/acel.12732 (PMC5847874; doi:10.1111/acel.12732)
Supplement: Supplementary file 1 [file ACEL-17-e12732-s001.pdf]

Supporting Information For:

Smurf2 regulates stability and the autophagic-lysosomal turnover of lamin A and its disease-associated form progerin

Aurora Paola Borroni, Andrea Emanuelli, Pooja Anil Shah, Nataša Ilić, Liat Apel-Sarid, Biagio Paolini, Dhanoop Manikoth Ayyathan, Praveen Koganti, Gal Levy-Cohen and Michael Blank

Supplementary Materials & Methods

Vectors and constructs

GFP- and MYC-Smurf2WT, mutant MYC-Smurf2(C716G) and HA-tagged ubiquitin were previously described (Blank *et al.*, 2012). N-terminal FLAG-tagged human lamin A and progerin were constructed by PCR, using the following primers: 5'-caccgaattcgagaccccgtcccagcggc-3' (forward primer containing EcoRI site) and 5'-atatgtcgacttacatgatgctgcagttctg-3' (reverse primer with SalI restriction site) using the template plasmids pBABE-puro-GFP-wt-lamin A and pBABE-puro-GFP-progerin, a gift from Tom Misteli (Addgene plasmids #17662 and #17663). The PCR products were digested with EcoRI and SalI and inserted into pRK2-FLAG vector. N-terminal mCherry-tagged human lamin A (mCherry-C1-lamin A) was constructed by similar procedure using another pair of primers: 5'-caccgaattcaatggagaccccgtcccagc-3' (forward primer with EcoRI site) and 5'-atatgtcgacttacatgatgctgcagttctg-3' (reverse primer containing SalI site). All constructs were full-sequence verified.

Cell transfections and generation of stable cell lines

cDNA transfections were performed using FUGENE 6 (Promega) according to the manufacturer's instructions. For siRNAs transfections, Oligofectamine (Invitrogen) was used. For Smurf2 overexpression in HGADFN167 and HGFDFN168 fibroblasts, cells were electroporated (Lonza VCA1001) with 5 μg of GFP-Smurf2 or GFP-empty vector, and analyzed 48 hrs after electroporation. For generating of Smurf2 stable knock-down, cells were infected with lentiviruses containing pLKO.1-Smurf2-puro vector (Sigma), and selected with puromycin for 2-3 weeks.

GST-fusion protein, pull-down assays and ubiquitination assays

GST fusion proteins were prepared from *E.coli* and isolated using Glutathione Sepharose 4B beads (GE Healthcare). Flag-lamin A and Flag-progerin were produced using TNT® SP6 Coupled Wheat Germ Extract System (L3260; Promega).

For the *in vitro* binding assay, Flag-tagged proteins were first pre-cleared with Glutathione-Sepharose beads for 1 hr at 4°C. After pre-clearing, these proteins were incubated with purified GST-Smurf2 or GST proteins in binding buffer. GST pull-down was conducted using Glutathione Sepharose 4B beads. Flag-progerin was pulled-down using agarose beads conjugated with FLAG

antibody (FLAG-M2 affinity gel; Sigma-Aldrich). Beads were then washed four times with ice-cold binding buffer, and proteins were eluted with 5X SDS sample buffer.

In vivo and *in vitro* ubiquitination assays were performed as previously described (Levy-Cohen *et al*., 2015; Emanuelli *et al*., 2017). In brief, for the *in vivo* ubiquitination assay cells were lysed with RIPA buffer supplemented with 5 mM NEM (N-Ethylmaleimide). Flag-lamin A and Flag-progerin were immunoprecipitated, and their ubiquitination pattern analyzed.

For the *in vitro* ubiquitination assay, Flag-lamin A and Flag-progerin derived from the TNT® reaction were incubated with 2 µg of GST or GST-Smurf2, 5 µg of HA-ubiquitin protein, E1 (UBE1; 100 ng), E2 enzyme (UbcH5c; 150 ng), and 100 mM ATP-Mg in the E3 ligase reaction buffer (BostonBiochem) for 2 hrs at 37°C. RIPA buffer was added to the reactions and Flag-lamin A and Flag-progerin were pulled down using M2-FLAG beads (Sigma).

qRT-PCR

Total RNA was extracted from Smurf2 knock-down and control MDA-MB-231 cells using RNeasy mini kit (Qiagen), according to the manufacturer's instructions. Total RNA was then reverse-transcribed with random primers using High Capacity cDNA Reverse Transcription Kit (Applied Biosystems). *LMNA* cDNA levels were determined using Fast SYBR Green Master mix and ViiA™ 7 Real-Time PCR System (Thermo Fisher Scientific). The experiments were performed three times with three technical replicates for each experiment. The gene expression was calculated using $2^{-\Delta\Delta Ct}$ method, and normalized to GAPDH gene. The following primers were used for lamin A/C expression analysis: Forward: 5'-aatgatcgcttggcggtctac-3' and Reverse: 5'-cttcttggtattgcgcgcttt-3'. Primers used for GAPDH: Forward 5'-ggagcgagatccctccaaaat-3' and Reverse 5'-ggctgttgtcatacttctcatgg-3'.

Nuclear circularity/deformability analysis

Nuclear circularity was measured as previously described (Goldman *et al*., 2004). Briefly, the circularity of the nucleus with a perfect circle shape was scored as 1.0. Nuclei with lobulation and/or blebbing were valued between >0 and <1.0, depending on the severity of the phenomena. The outline of the nuclei, obtained under confocal fluorescent microscope, was traced with a freehand selection tool, followed by the measurement of nuclear circularity with the ImageJ software (the software measures the roundness of the nucleus using the formula: $4\pi \times area/perimeter^2$).

Statistical analysis

Two-tailed student *t*-test was applied for statistical analysis of data. Data with *P*-values of less than 0.05 were considered statistically significant.

Supplementary Figures

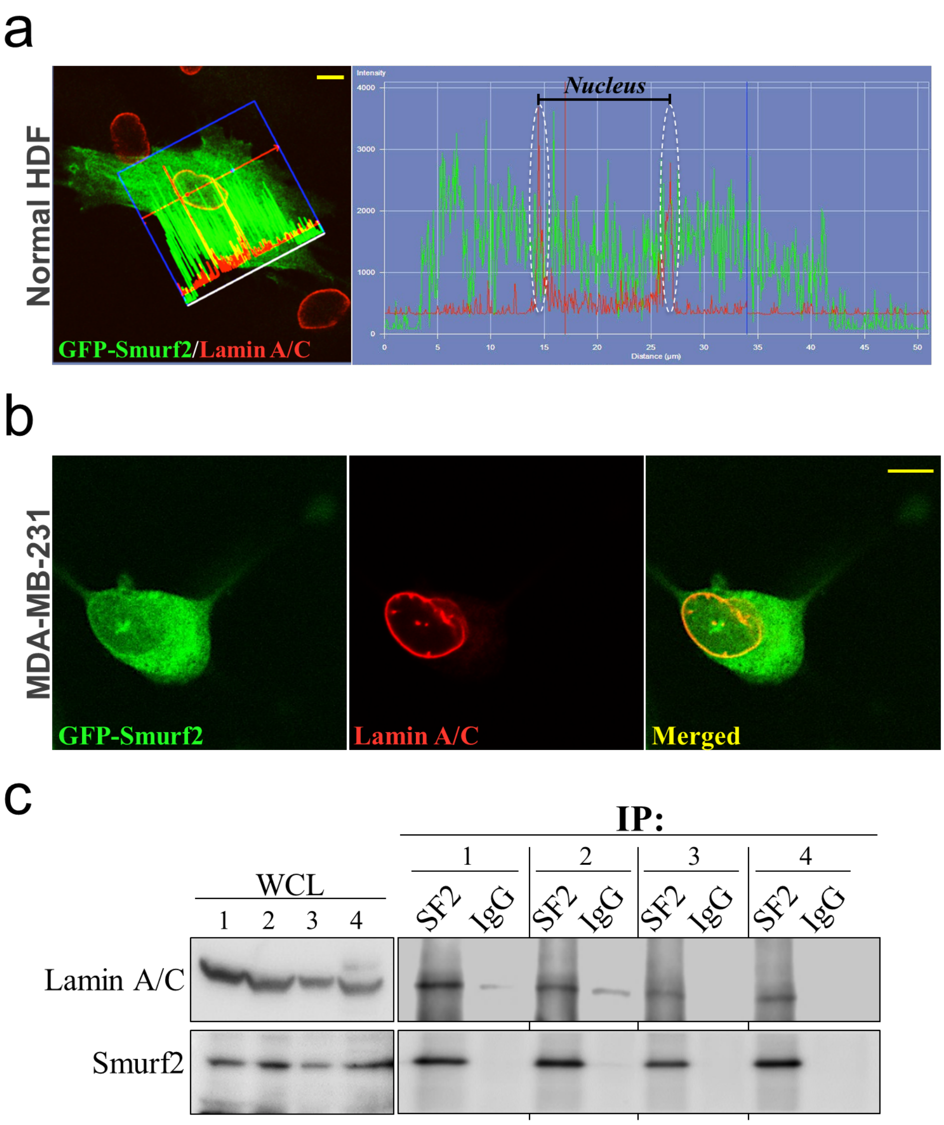

Figure S1. Smurf2 interacts with lamin A in different types of cells. (a) Confocal analysis showing co-localization of GFP-Smurf2 and lamin A/C on the nuclear envelope of normal HDF cells. Bars, 10 µm. (b) Co-localization of GFP-Smurf2 and lamin A/C in human breast adenocarcinoma MDA-MB-231 cells. Bars, 10 µm. (c) Co-immunoprecipitation (co-IP) between Smurf2 and A-lamins. HEK-293T cells were lysed using four different lysis buffers (containing different concentrations of detergents): #1–0.5%NP-40; #2–1%NP-40; #3–detergent-free lysis buffer, but containing 600 mM KCL (freezing-thawing buffer); #4–RIPA buffer containing a non-ionic detergent 1%NP-40, and two ionic detergents 0.1%SDS and 0.5%Sodium deoxycholate. Cell lysates were incubated with either anti-Smurf2 or normal rabbit IgG antibodies. Subsequently, co-IPs were washed, resolved in SDS-PAGE, and probed with anti-Smurf2 and anti-lamin A/C antibodies.

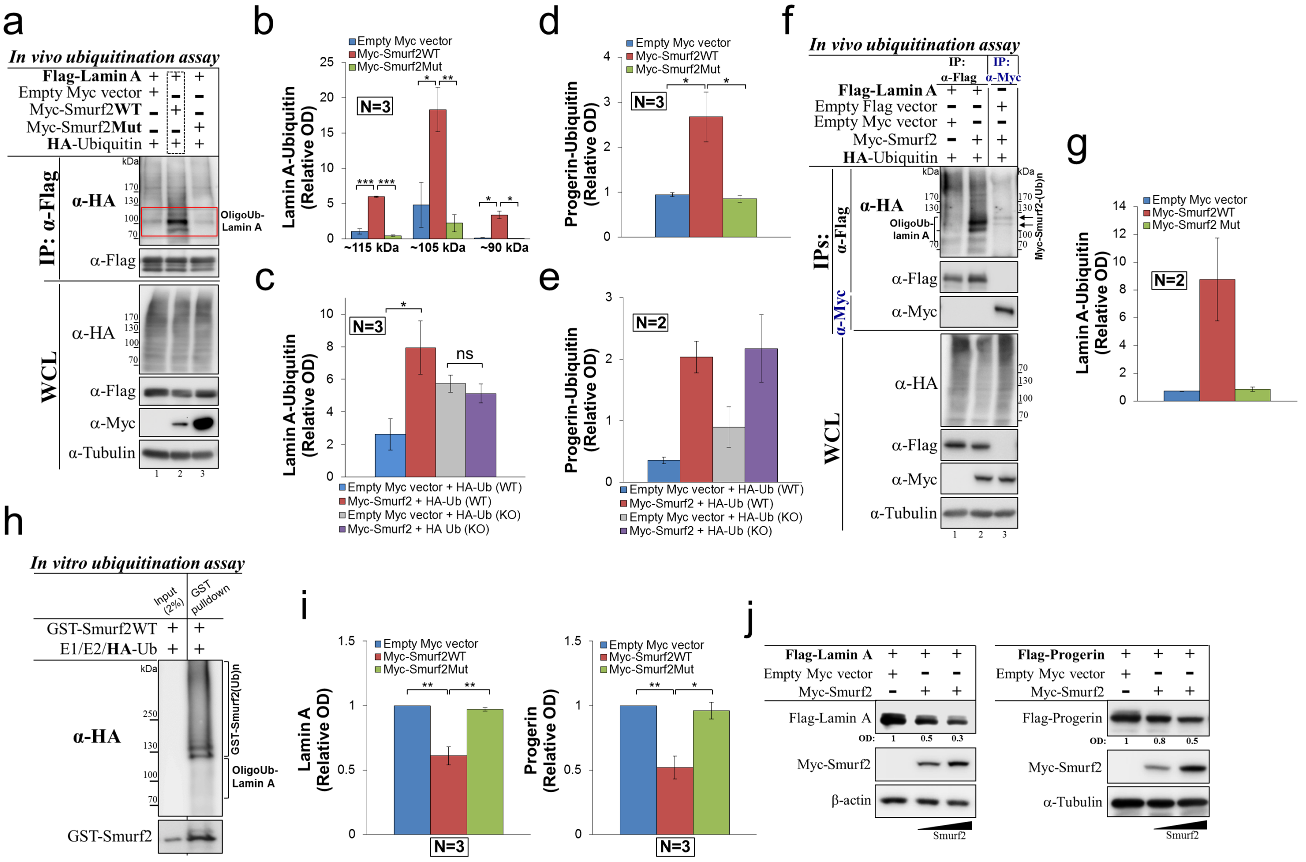

Figure S2. Smurf2 ubiquitinates lamin A and progerin and decreases the cellular levels of these proteins in a dose-dependent manner. (a) *In vivo/in cellulo* ubiquitination assay showing that Smurf2 ubiquitinates lamin A in catalytically-dependent manner. (b) Quantification of the western blot analysis data on Smurf2-mediated ubiquitination of lamin A obtained from three independent experiments (N=3). The intensity of ubiquitinated lamin A bands was normalized to the intensity of Flag-lamin A measured in the IP samples. Data are mean $\pm$ SEM. $*P < 0.05$, $**P < 0.01$, and $***P < 0.001$. (c) Quantification of the western blot analysis data on Smurf2-mediated oligo-ubiquitination of lamin A (described in Fig. 2b). Data are mean $\pm$ SEM of three independent experiments. $*P < 0.05$; NS – non-significant. (d) Quantification of the western blot analysis data on Smurf2-mediated ubiquitination of Flag-Progerin expressed in HEK-293T cells and described in Fig. 2c. The intensity of the progerin ubiquitination was normalized to the intensity of Flag-Progerin measured in the IP samples. Data are mean $\pm$ SEM of three independent experiments. $*P < 0.05$. (e) Quantification of the western blot analysis data on Smurf2-mediated multi-ubiquitination of progerin obtained in two independent experiments. (f) *In vivo/in cellulo* ubiquitination assay showing that the ubiquitination pattern of lamin A is distinct from Smurf2 ubiquitination/autoubiquitination. Flag-lamin A was immunoprecipitated from the reaction with anti-FLAG antibody; Myc-Smurf2 was IPed using anti-Myc antibody. The ubiquitination patterns of lamin A and Smurf2 were analyzed using anti-HA-ubiquitin antibody. (g) Quantification of lamin A *in vitro* ubiquitination. (h) Western blot analysis of Smurf2 *in vitro* auto-ubiquitination. GST-Smurf2 was pulled down from the reaction using GST-beads. This experiment was conducted concurrently with *in vitro* ubiquitination experiments of lamin A and progerin shown in Fig. 2e,f. (i) Quantification of the western blot analysis data on Smurf2-mediated degradation of lamin A and progerin expressed in HEK-293T cells, and its dependence on Smurf2 E3 ubiquitin ligase functions. Data are mean $\pm$ SEM of three independent experiments. $*P < 0.05$, $**P < 0.01$. (j) Western blot analysis showing that Smurf2 overexpression reduces the levels of Flag-lamin A and Flag-Progerin expressed in HEK-293T cells in Smurf2 dose-dependent manner.

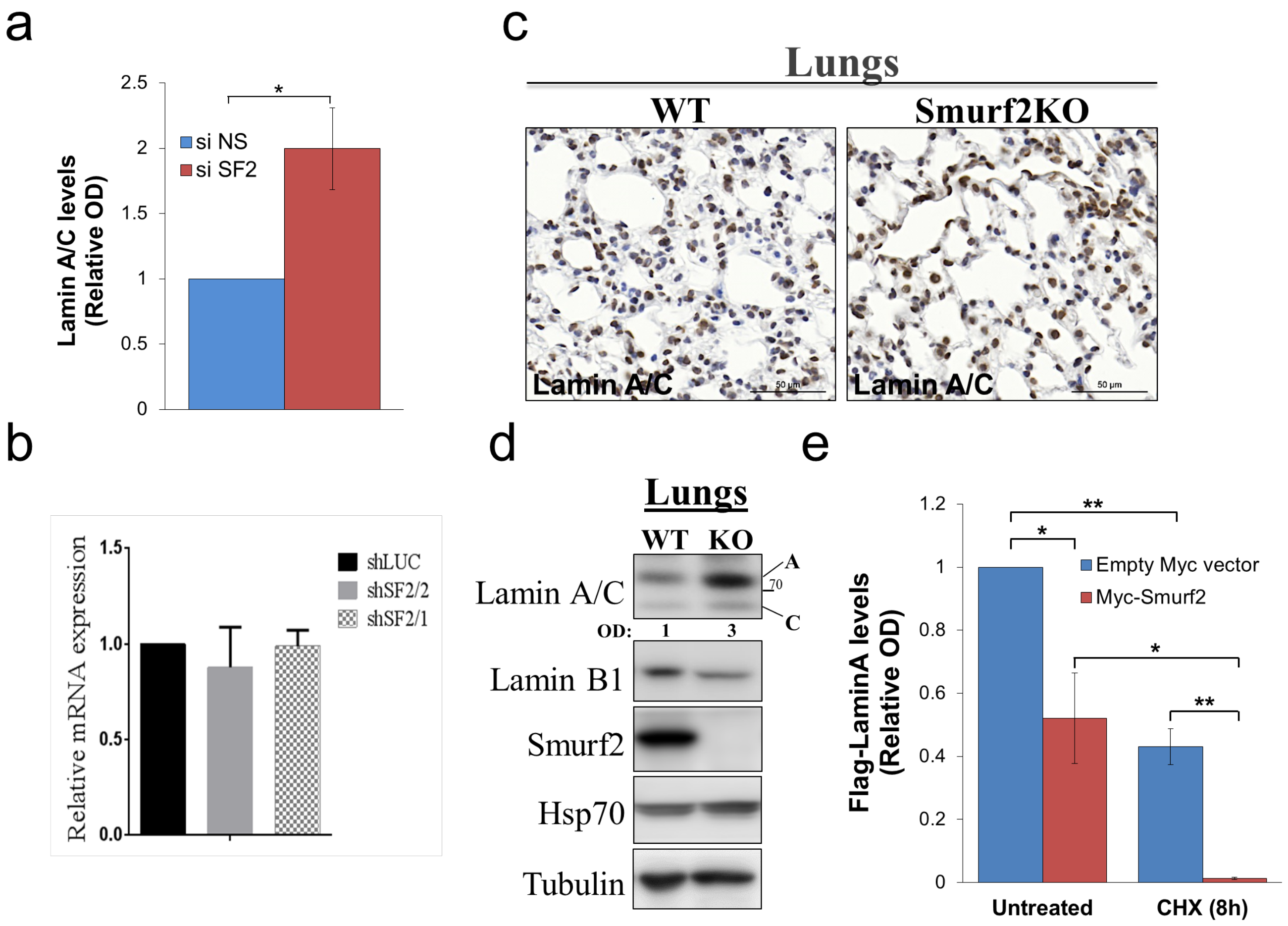

Figure S3. Smurf2 regulates A-lamins post-translationally. (a) Quantification of effects of acute Smurf2 knockdown using siRNA on the lamin A/C levels in MDA-MB-231 cells. Data are mean ± SEM (N=2). $*P < 0.05$. (b) qRT-PCR analysis of lamin A/C mRNA levels in Smurf2 knock-down and control MDA-MB-231 cells. Data are represented as mean ± SD of three independent experiments with three technical replicates per experiment. Lamin A/C gene expression was normalized to GAPDH gene. shSF2/1 and shSF2/2 are two distinct shRNAs used for Smurf2 knock-down. shLuc, cell knocked-down with shRNA against luciferase (control). (c) IHC staining of lamin A/C in Smurf2WT and KO lung tissues. The nuclei were counterstained with hematoxylin (blue). Bars, 50 µm. (d) Western blot analysis of lamins levels in lung tissues of Smurf2KO and wild-type control animals. (e) Quantification of Western blot analyses on the effects of Smurf2 overexpression on Flag-lamin A in CHX-treated and untreated HEK-293T cells. Data are mean ± SEM (n=3). $*P < 0.05$; $**P < 0.01$.

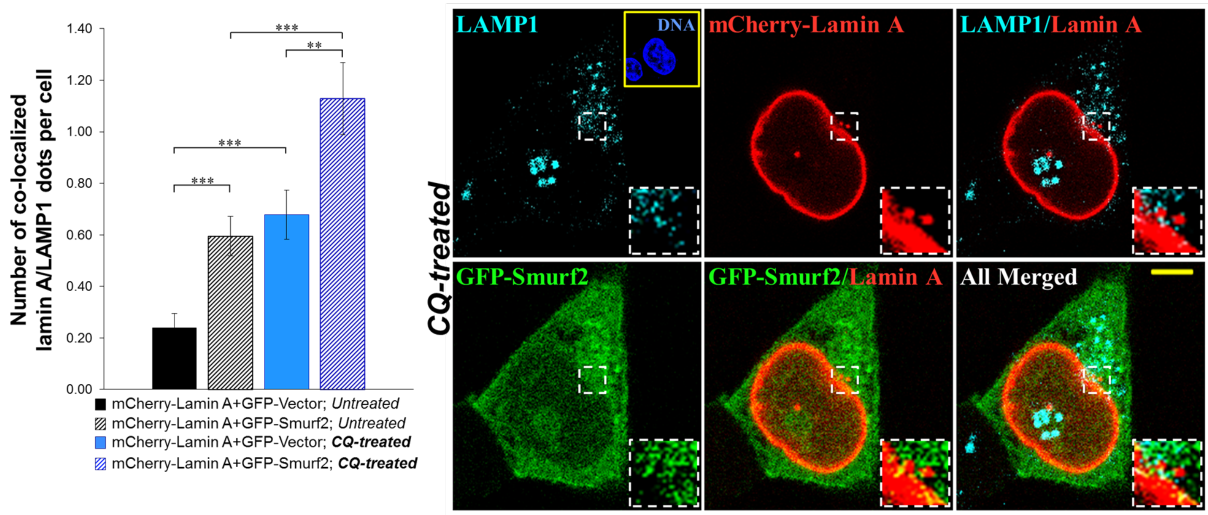

Figure S4. Smurf2 overexpression triggers lamin A association with the lysosomal protein LAMP1. HEK-293T cells co-expressing mCherry-lamin A and GFP-Smurf2 (or an empty GFP vector) were immunostained with anti-LAMP1 antibody, and the co-localization between these proteins in untreated and chloroquine-treated cells was visualized under confocal microscope. Left panel shows quantification of the confocal data obtained in two independent experiments with an average of 96 cells/group. Data are mean ± SEM. $^{**}P < 0.01$, $^{***}P < 0.001$. Representative confocal images, indicating the association sites of mCherry-lamin with LAMP1 in Smurf2-overexpressing cells, are shown on the right. Bars, 10 μm.

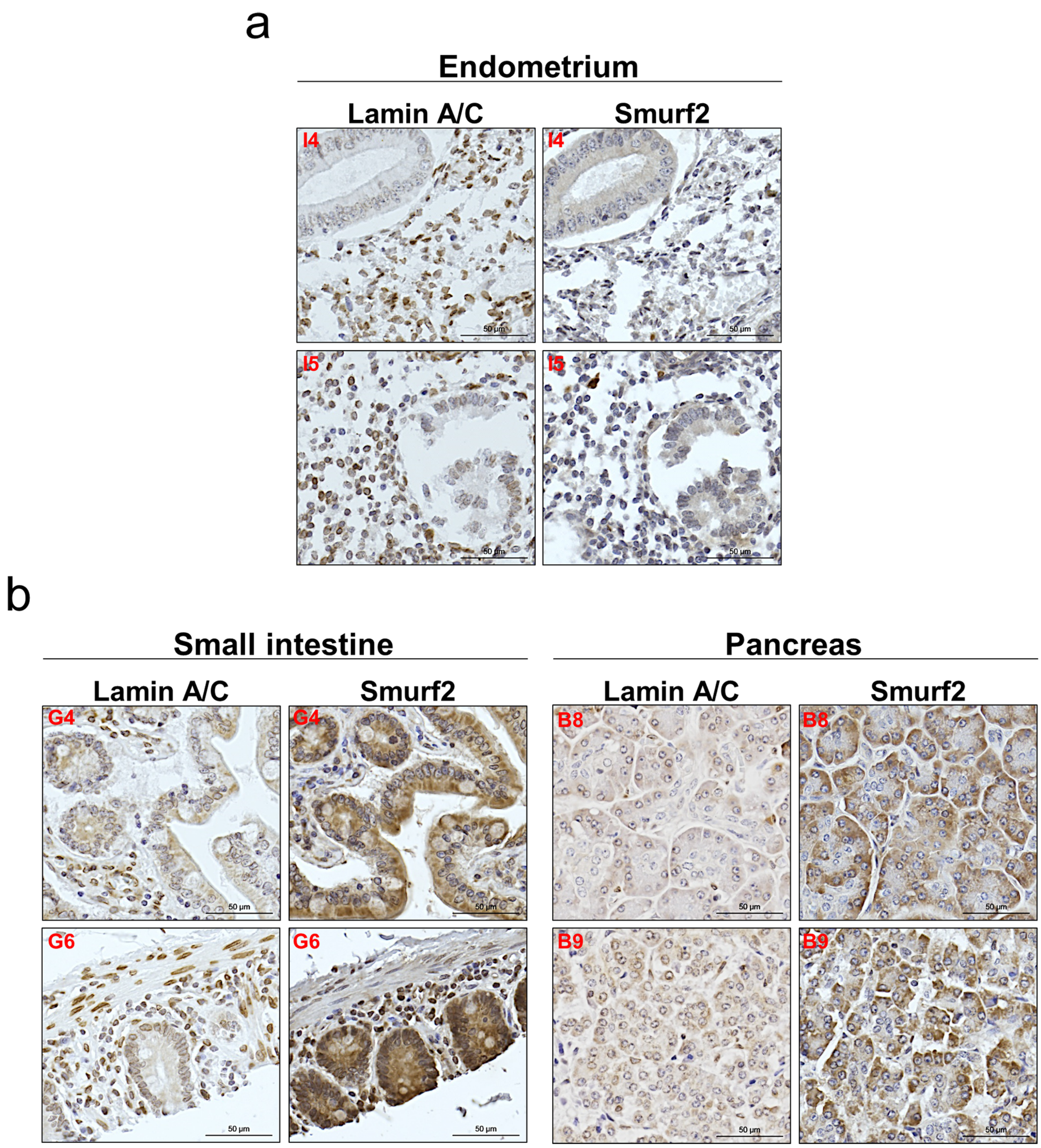

Figure S5. Inverse relationship between Smurf2 and A-lamins in human normal tissues. (a) IHC staining showing low expression levels of Smurf2 and higher expression levels of lamin A/C in endometrial tissues. Bars, 50 µm. (b) IHC staining showing higher expression levels of Smurf2 and lower expression levels of lamin A/C in small intestine and pancreatic tissues. Bars, 50 µm.

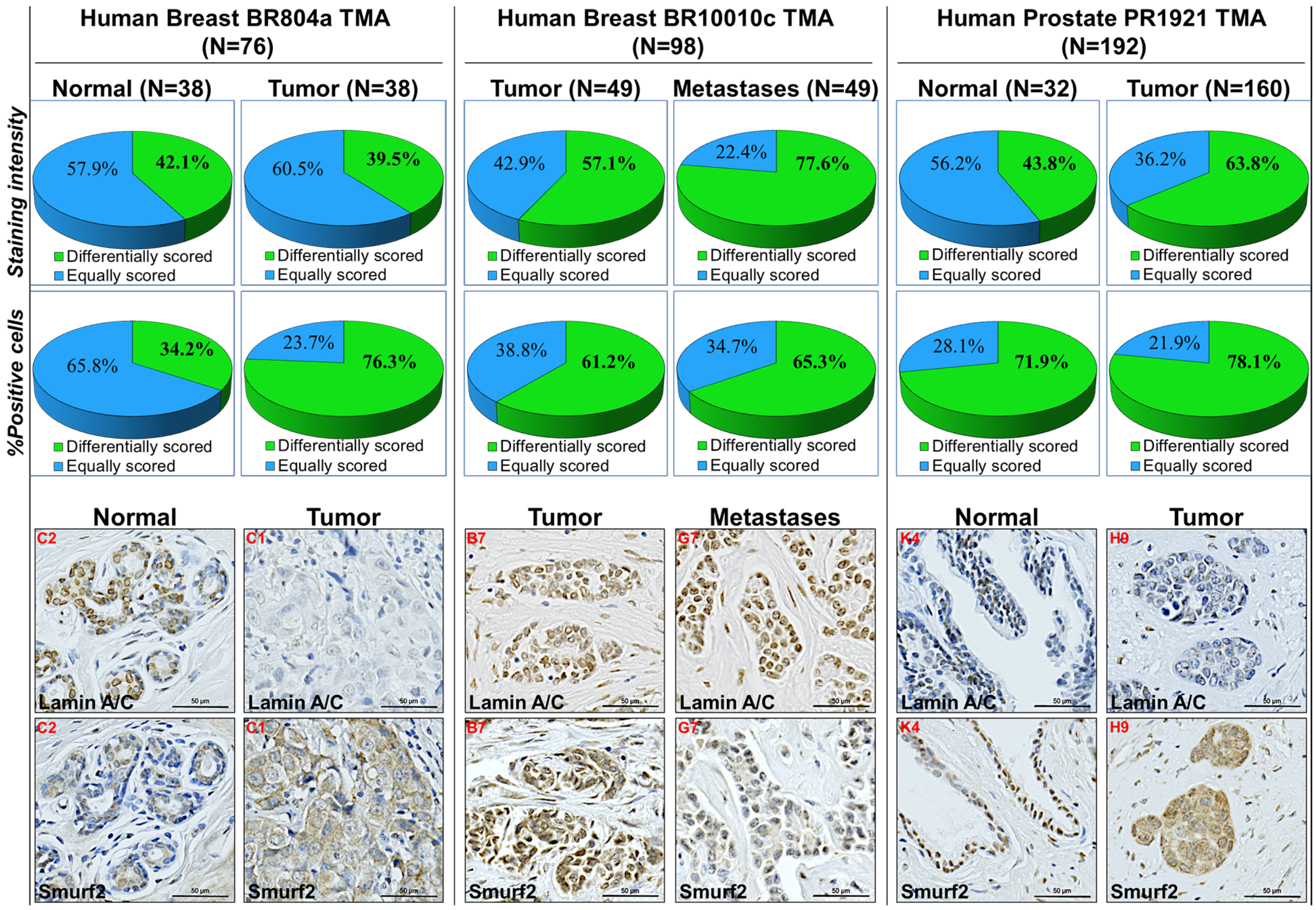

Figure S6. Reciprocal relationship of Smurf2 and A-lamins in human cancer TMAs.
Two human breast cancer TMAs and prostate carcinoma TMA, containing normal, primary tumor and metastatic tumor tissues, were stained for Smurf2 and lamin A, and scored for the staining intensity and percentage of positive cells. Representative images showing differential expression of Smurf2 and lamin A in the same tissue samples are shown on the bottom of the figure. Bars, 50 μm.

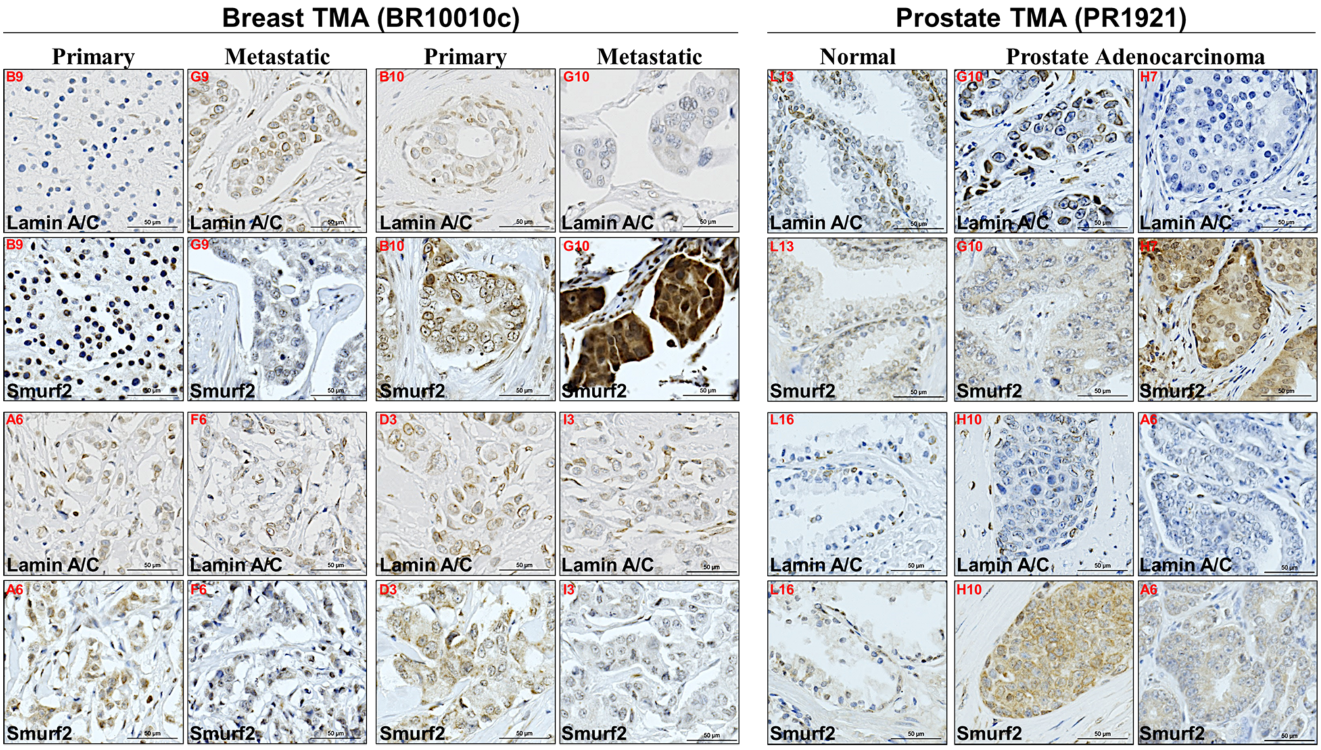

Figure S7. Representative IHC images showing inverse relationship between Smurf2 and A-lamins in human breast and prostate cancer tissues. Note, despite the heterogeneous expression of Smurf2 and lamin A/C in cancer tissues, higher expression levels of Smurf2 showed lower expression of lamin A/C, and *vice versa*. Scale bars, 50 µm.
